# The role of core and accessory type IV pilus genes in natural transformation and twitching motility in the bacterium *Acinetobacter baylyi*

**DOI:** 10.1371/journal.pone.0182139

**Published:** 2017-08-03

**Authors:** Colleen G. Leong, Rebecca A. Bloomfield, Caroline A. Boyd, Amber J. Dornbusch, Leah Lieber, Flora Liu, Amie Owen, Erin Slay, Kristine M. Lang, C. Phoebe Lostroh

**Affiliations:** 1 Department of Molecular Biology, Colorado College, Colorado Springs, Colorado, United States of America; 2 Department of Physics, Colorado College, Colorado Springs, Colorado, United States of America; Centre National de la Recherche Scientifique, Aix-Marseille Université, FRANCE

## Abstract

Here we present an examination of type IV pilus genes associated with competence and twitching in the bacterium *Acinetobacter baylyi* (strain ADP1, BD413). We used bioinformatics to identify potential competence and twitching genes and their operons. We measured the competence and twitching phenotypes of the bioinformatically-identified genes. These results demonstrate that competence and twitching in *A*. *baylyi* both rely upon a core of the same type IV pilus proteins. The core includes the inner membrane assembly platform (PilC), a periplasmic assemblage connecting the inner membrane assembly platform to the secretin (ComM), a secretin (ComQ) and its associated pilotin (PilF) that assists with secretin assembly and localization, both cytoplasmic pilus retraction ATPases (PilU, PilT), and pilins (ComP, ComB, PilX). Proteins not needed for both competence and twitching are instead found to specialize in either of the two traits. The pilins are varied in their specialization with some required for either competence (FimT) and others for twitching (ComE). The protein that transports DNA across the inner membrane (ComA) specializes in competence, while signal transduction proteins (PilG, PilS, and PilR) specialize in twitching. Taken together our results suggest that the function of accessory proteins should not be based on homology alone. In addition the results suggest that in *A*. *baylyi* the mechanisms of natural transformation and twitching are mediated by the same set of core Type IV pilus proteins with distinct specialized proteins required for each phenotype. Finally, since competence requires multiple pilins as well as both pilus retraction motors PilU and PilT, this suggests that *A*. *bayl*yi employs a pilus in natural transformation.

## Introduction

Natural transformation is the ability of physiologically competent bacteria to internalize exogenous DNA and incorporate it into their own genomes. Twitching is a method of motility in which bacteria extend pili, attach to a surface using the distal end of the pili, then retract the pili thereby pulling themselves toward the attached tips of the pili. In many species, natural transformation and twitching are mediated by type IV pili.

Type IV pili are nanomachines with extracellular, transenvelope, and cytoplasmic components (recently reviewed in [1, 2]). The extracellular fiber is between 6–9 nm in diameter, can be many times longer than a cell, and in most species can be extended and retracted. The extracellular fiber is comprised primarily of subunits known a pilins. While the N-terminal region of all pilins is highly conserved, the rest of any given pilin can vary wildly and these variations are associated with differences in pilin function (reviewed in [2]). The identity of which pilins comprise the fiber may also vary in differing machines or organisms. In some systems, the fiber’s distal terminal subunit is not a pilin but an adhesin, which is not structurally related to pilins [3–8]. The pilus is assembled in the envelope by a machine consisting of an assembly platform in the inner membrane, a cytoplasmic assembly ATPase (motor), and a prepilin peptidase [9–11]. There is also at least one cytoplasmic retraction ATPase [11]. The pilus shaft exits the periplasm of a Gram negative cell through an aqueous pore in the outer membrane formed by a secretin protein [12–15], which in turn is stabilized by another outer membrane protein known as a pilotin [16]. A transenvelope pilus alignment complex with cytoplasmic and periplasmic components stabilizes this multiprotein arrangement and also plays a dynamic role during extension and retraction [17–22].

The various components of a Type IV pilus machine are used in a similar manner in the mechanisms of both competence and twitching. In the case of twitching, the bacteria extend a twitching pilus. In contrast to the known mechanism of twitching, the use of a pilus during natural transformation is not as clear, although recent work has shed light on this issue. It is known that competence in most organisms requires type IV pilus proteins including pilins, the secretin, and the motor proteins [23]. Evidence regarding the role of an actual extracellular pilus fiber in *Neisseria*, however, is mixed. *Neisseria* uptakes only species-specific DNA that contains repeated DUS sequences. For many years it was observed that the *Neisseria* type IV pilus does not bind preferentially to DNA with DUS sequences, suggesting that in *Neisseria* there is no competence associated pilus [24]. But more recently a minor pilin in the *Neisseria* pilus has been shown to bind better to DNA with DUS sequences than to DNA lacking them [25, 26]. While a model to explain these complex observations in *Neisseria* has not emerged uncontested, a competence pilus has now been reported in other species such as *Streptococcus pneumoniae* and *Vibrio cholerae* [27–29]. Thus it now seems that in at least some species there is a competence pilus that cells extend that binds to extracellular DNA in order for transformation to occur (recently reviewed in [23,30]).

Once the pilus has encountered its target, DNA for transformation or a surface for twitching, there must be some mechanism to bind the pilus to its respective target. For transformation, in cases where it is known that the pilus binds to DNA, the components of particular pili that bind to DNA are species-specific and, when known, are usually pilin subunits [23–27, 29–32]. In an analogous manner, for twitching it may be an adhesin protein or particular pilin at the end of the pilus that allows it to attach to the surface, or there may be several sites of binding between a twitching pilus and the target surface (reviewed in [2, 23]).

After the pilus has attached to DNA for transformation or to a surface for twitching, in both cases dedicated ATPase retraction motor proteins reel in the pili (recently reviewed in [2, 33, 34]). For transformation in broth, pilus retraction likely moves the DNA to the cell’s surface and for twitching, retraction pulls the cell forward. Because in one case the pilus is pulling only the weight of DNA while in the other it is pulling on the weight of the cell, these two behaviors may require different amounts of force to be exerted by the motors and different physical properties (such as elasticity) of the pilus. Some strains of *Acinetobacter baumannii* are exclusively competent during twitching on a surface, so there may be cases where pilus retraction drags the cell forward, fortuitously allowing it to collide with DNA in the surrounding environment [35].

For both twitching and natural transformation, the pilus protrudes through an aqueous channel formed by a secretin located in the cell’s outer membrane [2, 13]. For transformation the DNA enters the periplasm through the secretin pore. Now speaking only of transformation, the mechanism by which DNA enters the periplasm in both *Neisseria* and *Vibrio* appears to be biased diffusion facilitated by a periplasmic DNA binding protein [28, 36, 37]. After crossing the outer membrane, one strand of the DNA is degraded by an exonuclease while the other passes through a dedicated inner membrane transporter. Once inside the cytoplasm, the single strand of DNA is either degraded or recombined with DNA already resident in the cytoplasm.

Although Type IV pili are required for both natural transformation and twitching and Type IV pili genes are present in many bacterial species, these species do not all share the same competence and twitching phenotypes. *Neisseria* is both competent and twitches and these phenotypes are both known to require many of the same type IV pilus genes in *Neisseria* [24]. Recently, other model organisms with type IV pilus genes (*Myxococcus xanthus*) that were once studied to understand twitching motility or biofilm formation were discovered to also be competent [34, 38–42]. In contrast, *Pseudomonas aeruginosa* is a well-studied model that appears to twitch but is not competent [33, 43]. The lack of competence is probably not due to absent pilins; indeed PilA can bind to DNA, but model strains such as PAO1 do not appear to encode proteins for transporting DNA through the periplasm and inner membrane [32, 44]. Since many bacteria have type IV pilus homologs, including all those mentioned above, it remains an open question whether such bacteria will be found to be both competent and twitch, and if not, why not? Perhaps there are core type IV pilus proteins required for both competence and twitching, while the presence or absence of accessory proteins specialized for one or the other determine physiology.

*Acinetobacter baylyi* is a bacterium that can help address these questions. *A*. *baylyi* strain ADP1 (ATCC33305, BD413) is a non-pathogenic model for the Gram negative *Acinetobacter* genus, comprised of aerobic soil organisms that are tolerant of wide temperature ranges and desiccation, and can use a diverse array of carbon sources (reviewed in [45, 46]). ADP1 is ideal for studying the genetic link between twitching motility and competence primarily because the species is both very good at DNA acquisition and very motile under the same nutritional conditions. As an example of *A*. *baylyi’s* competence, every cell in the population imports more than 60 different molecules of DNA at peak transformation efficiency [47]. In terms of twitching, as reported herein, ADP1 twitch zones on soft agar are large and therefore accurately measured. In addition *A*. *baylyi’s* entire genome is sequenced, and a full library of single-gene non-polar deletion mutants of ADP1 is available, thereby facilitating its use in studies of the genetic basis of competence and twitching.

Despite these advantages, genetic connections between twitching and competence have not been previously reported in *A*. *baylyi*. Twitching motility was actually first discovered through study of *Acinetobacter* in 1961 [48, 49]. Since the discovery of competence in *A*. *baylyi* in 1969, about a dozen genes were tested for their effect on competence and twitching, but none of them were found to affect both phenotypes (reviewed in [50]). However, no studies have tested a comprehensive set of predicted type IV pilus genes for their effect on competence and twitching in ADP1.

Here we present such an examination of ADP1 type IV pilus operons associated with competence and twitching. We began with a bioinformatics analysis to identify potential competence and twitching genes and their operons in ADP1. We found that the operons are scattered throughout the chromosome and that some of those operons contain essential genes. For each of the identified operons, we measured the competence and twitching phenotypes of at least one non-polar single-gene knockout mutant. We found that in *A*. *baylyi* competence and twitching both rely upon a core of the same type IV pilus proteins; however there are also proteins, including pilins, specialized in one functionality or the other. These findings suggest that the mechanism of natural transformation is likely very similar to that of twitching and that the function of accessory proteins in any given organism may not be predictable based on homology alone.

## Materials and methods

### Bioinformatic analysis

We used the curated Kyoto Encyclopedia of Genes and Genomes (kegg.jp) database to search for ADP1 homologs of type IV pilus proteins from *Neisseria gonorrhoeae* FA 1090, *Pseudomonas aeruginosa* PAO1, and *Vibrio cholerae* N16961 [51]. We used the ComEC and ComEA sequences from *N*. *gonorrhoeae* and *V*. *cholerae* to identify ADP1 periplasmic or inner membrane DNA transport proteins. We used the *P*. *aeruginosa* twitching PilR, PilS, PilG, PilH, PilI, PilJ, and ChpA signal transduction proteins to find ADP1 homologs. We used an algorithm to predict operon composition in ADP1 (http://meta.microbesonline.org/operons/gnc62977.html; [52, 53]. We used Kyoto Encyclopedia of Genes and Genomes software for analysis of protein motifs and evolutionary history [51]. Multiple alignments were performed using CLUSTAL-Ω [54]. Proteins were analyzed using Pfam [55, 56] made available through KEGG. Pairwise alignments were performed using EMBOSS [57].

### Bacteria and media

We used an *A*. *baylyi* strain from the American Type Culture Collection (ATCC) strain 33305/ADP1/BD413. We rehydrated ADP1 from the ATCC on minimal media plates with recipe: 25 ml 0.5 M KH_2_PO_4_, 10 ml 10% (NH_4_)_2_SO_4_, 1 ml concentrated base, 3.35 g Na_2_HPO_4_, 18 g BD Bacto^TM^ Agar, 10 ml of 1 M succinate, and QS to 1 L with dH_2_O. Concentrated base recipe: Dissolved 20 g nitrolotriacetic acid and 14.6 g KOH in dH_2_O. Added 28.9 g anhydrous MgSO_4_, 6.67 g CaCl_2_-2H_2_O, 0.019 g (NH_4_)_6_Mo_7_O_24_-4H_2_O, 0.198 g FeSO_4_-7H_2_O, and 100 ml of “Metals 44” (2.5 g EDTA free acid, 10.95 g ZnSO_4_-7H_2_O, 1.54 g MnSO_4_-H_2_O, 0.392 g CuSO_4_-5H_2_O, 0.250 g C(NO_3_)_2_-6H_2_O, 0.177 g Na_2_B_4_O_7_-10H_2_O, 100 μL H_2_SO_4_ dissolved in 1 L dH_2_O). The pH was adjusted to 6.8 with up to 100 ml of 1 M KOH in dH_2_O and added dH_2_O to a total volume of 1 L.

We prepared LB using a recipe of 10 g tryptone, 5 g yeast extract, and 10 g NaCl per liter of H_2_O. Frozen stocks consisting of LB with 25% glycerol were prepared from rehydrated streak plates and used for no longer than two years from the date of cryopreservation. Starting with the cryogenically preserved cells, we made streak plates and used colonies from them to inoculate broths. Streak plates kept at 4°C were used for up to seven days and then discarded. Plates contained 1.5% agar unless they were soft agar used specifically for twitching assays.

### Strain construction

Gene knockouts in which a *tdk-kan* cassette replaces the coding sequence were obtained from the Genoscope collection [45]. Before testing the effects of mutations they were moved into wild type cells from ATCC (ATCC33305/ADP1/BD413). A strain table is found in the supplementary materials. To move the mutations into the ATCC33305 background, crude lysates from the knockout collection were prepared by growing overnight cultures in LB supplemented with kanamycin (10 μg/mL) at 37C with high aeration. The overnight cultures were pelleted and 1.5 ml of cells were resuspended in 100 μL of sterile water and heated at 95°C in a heat block for 2 hours to lyse the cells. We plated 5 μl samples of each lysate to assess sterility. The lysates were used to transform wild type cells to kanamycin resistance. Location of the mutation was verified by PCR using published P7 and P8 primers; strains giving unexpected amplicons were not used in further analysis. Attempts were made to complement the mutants by cloning *pilX*, *comE*, *fimT*, and *pilV* using a variety of single-copy strategies but were unsuccessful, likely due to toxicity [58, 59], and thus no further complementation was attempted. In support of the toxicity hypothesis, *fimT* could be cloned when its expression was extremely depressed through the use of an especially poor ribosome binding site or replacing the start codon with GUG (data not shown).

### Donor DNA isolation for transformation assays

To use as donor DNA for transformation assays, we isolated chromosomal DNA from a spontaneous streptomycin-resistant ADP1 strain provided by Bruce Voyles (Grinnell College). After culturing these str^R^ cells in 12 ml of LB-supplemented with 20 μg/ml streptomycin (str^20^) broth overnight with high aeration at 37°C, we pelleted the cells and resuspended them in 1 ml of 1% sterile saline. We split the total volume into two microfuge tubes and added 500 μl of phenol-chloroform-isoamyl alcohol (25:24:1) to each tube. After vortexing to mix and microfuging to separate the organic and aqueous phases, the aqueous phase was removed to a new microfuge tube, and the DNA precipitated using standard methods with sodium acetate and ethanol. The DNA that originated from 12 ml of cells was resuspended in a final volume of 100 μl in EB buffer (Qiagen), composed of 10 mM Tris-Cl (pH = 8.5). A Nanodrop spectrophotometer indicated a final concentration of about 1 mg ml^-1^ for each preparation with a typical A_260_/A_280_ of 1.6 for different preparations of chromosomal DNA.

### Measuring natural transformation efficiency

We grew wild type and mutant cells overnight at 37°C with high aeration in LB or LB supplemented with kanamycin (10 μg ml^-1^), respectively. The next day we mixed 5 μL of DNA from a streptomycin-resistant mutant (1 mg ml^-1^) with 50 μL of an overnight culture and then transferred the 55 μL to the center of an LB plate, forming a puddle. After overnight incubation at 37°C, the next day we scraped the cells in each puddle into 750 μL of saline (10 g L^-1^ NaCl), pipetting and vortexing to mix thoroughly. We performed a ten-fold dilution series from 10^0^−10^7^ in a microtiter plate, diluting with saline (10 g L^-1^ NaCl). For each independent trial, we plated two or three ten μl spots of each dilution on LB agar to obtain total CFU ml^-1^ and on LB agar supplemented with streptomycin (20 μg ml^-1^) to obtain transformed CFU ml^-1^. Transformation efficiency is defined as transformed CFU ml^- 1^/total CFU ml^-1^. The transformation efficiency of every mutant was determined using three independent trials on three separate days. We never observed any spontaneous streptomycin-resistant colonies from multiple control 10 μl spots of wild type cells plated on streptomycin for any trial.

### Measuring twitching zones

We grew wild type and mutant cells overnight at 37°C with high aeration in LB or LB supplemented with kanamycin (10 μg/mL), respectively. The next day we applied 4 μL of overnight cells to one quadrant of an LB soft agar plate (0.5% agar) containing 0.01% triphenyltetrazolium chloride, a redox sensitive dye that turns red when oxidized by respiration. For every trial we always had two wildtype spots. We incubated the plates in an incubator at 37°C for 4–6 hours until wild type twitching zones achieved a minimum diameter of 17 mm. When necessary because of prevailing meteorological conditions, a humidified incubator was used because low agar plates dehydrate faster than 1.5% agar plates. We did not use data from trials with wild type growth below this minimum. After the twitching period, we refrigerated plates overnight to allow the cells to become deep red. We measured the mutant and both wild type twitching diameters. We define the twitching ratio as mutant diameter/wild type diameter. We obtain two such ratios for each trial, one from each of the two wild type measurements, and we average the two ratios to obtain one twitching measurement. At least two independent twitching measurements were obtained for every mutant and for all but three mutants, six or more independent measurements were obtained.

## Results

### Candidate competence and twitching proteins in ADP1

Twitching is known to originate in type IV pili in many organisms. In addition, in ADP1 several type IV pilus genes are required for competence [50]. Motivated by this knowledge, we started with known type IV pilus protein sequences from *Neisseria gonorrhoeae*, *Pseudomonas aeruginosa*, and *Vibrio cholerae*. We used these sequences to search for the most similar protein encoded by the ADP1 genome in the Kyoto Encyclopedia of Genes and Genomes. By the same method, we also identified homologs of two other proteins, ComEA (ACIAD3064) and ComA (ACIAD2639), that are needed for competence in *N*. *gonorrhoeae* and *V*. *cholerae* for DNA transport (reviewed in [60, 61]). Finally we sought homologs of signal transduction proteins that affect twitching in *P*. *aeruginosa* [62].

Table 1 lists the ADP1 type IV pilus proteins together with their homologs in the other bacteria and the known functionalities of the proteins in the other bacteria. Table 2 lists other candidate ADP1 competence and twitching proteins identified with their homologs in the other bacteria. Tables 1 and 2 thereby present a comprehensive list of the type IV pilus, DNA transport, and signal transduction proteins likely to be responsible for competence and/or twitching in ADP1.

**Table 1:** Type IV pilus protein homologs in ADP1.

Protein name in ADP1	ACIAD	*Neisseria gonorrhoeae* FA 1090	*Pseudomonas aeruginosa* PAO1	*Vibrio cholerae* N16961	Function	ADP1 Operon	Essential Genes in ADP1 Operon
PilD	360	PilD/1670^a^	PilD/4528^b^	PilD/2426^c^	Prepilin peptidase	362–359	359 & 362
PilC	361	PilG/1669	XcpS/3102	PilC/2425	Assembly platform	362–359	359 & 362
PilB	362	PilF/1673	PilB/4526	PilB/2424	Assembly ATPase	362–359	359 & 362
PilF	558	PilF/595	PilF/3805	?/1612	Secretin stability	557–564	561 & 562
FimT	695	none	FimT/4549	none	Pilin	695	none
PilU	911	PilT/346	PilU/396	PilT/463	Retraction ATPase	912–911	none
PilT	912	PilT/1908	PilT/395	PilT/462	Retraction ATPase	912–911	none
ComF	3314	PilE/1441	PilE/4556	PilE/857	Pilin	3321–3314	none
ComE	3315	PilE/1441	PilE/4556	PilE/857	Pilin	3321–3314	none
ComC	3316	PilC/55	PilY1/4554	none	Adhesin	3321–3314	none
PilX	3317	none	none	none	Pilin	3321–3314	none
ComB	3318	none	PilW/4552	none	Pilin	3321–3314	none
PilV	3319	none	PilV/4551	none	Pilin	3321–3314	none
FimU	3321	none	FimU/4550	FimT/858	Pilin	3321–3314	none
ComP	3338	PilE/1441	PilA/4525	PilA/2423	Pilin	3338–3337	3337 unknown
ComQ	3355	PilQ/94	PilQ/5040	PilQ/2630	Secretin	3360–3352	3353 & 3354
ComL	3356	PilP/95	PilP/5041	PilP/2631	Pilus assembly complex	3360–3352	3353 & 3354
ComO	3357	PilO/96	PilO/5042	PilO/2632	Pilus assembly complex	3360–3352	3353 & 3354
ComN	3359	PilN/97	PilN/5043	PilN/2633	Pilus assembly complex	3360–3352	3353 & 3354
ComM	3360	PilM/98	PilM/5044	PilM/2634	Pilus assembly complex	3360–3352	3353 & 3354

^a^Prefix to the protein numbers is "ngo_" as in "ngo_1670."

^b^Prefix to the protein numbers is "PA" as in "PA4528."

^c^Prefix to the protein numbers is "VC" as in "VC2426."

**Table 2:** DNA transport and twitching pilus signal transduction homologs in ADP1.

Protein name in ADP1	ACIAD	*Neisseria gonorrhoeae* FA 1090	*Pseudomonas aeruginosa* PAO1	*Vibrio cholerae* N16961	Function	ADP1 Operon
DNA transport						
ComA	2639	ComA/1879^a^		ComEC/1879 ^c^	DNA transport	2639
ComEA	3064	ComE/1178		ComEA/1917	DNA transport	3065–3064
Twitching signal transduction						
PilR	258		PilR/4547^b^		Signal transduction	260–258
PilS	259		PilS/4546		Signal transduction	260–258
PilG	786		PilG/408		Signal transduction	786–790
PilH	787		PilH/409		Signal transduction	786–790
PilI	788		PilI/410		Signal transduction	786–790
PilJ	789		PilJ/411		Signal transduction	786–790
ChpA	790		ChpA/413		Signal transduction	786–790

^a^Prefix to the protein numbers is "ngo_" as in "ngo_1670."

^b^Prefix to the protein numbers is "PA" as in "PA4528."

^c^Prefix to the protein numbers is "VC" as in "VC2426."

**Fig 1:**
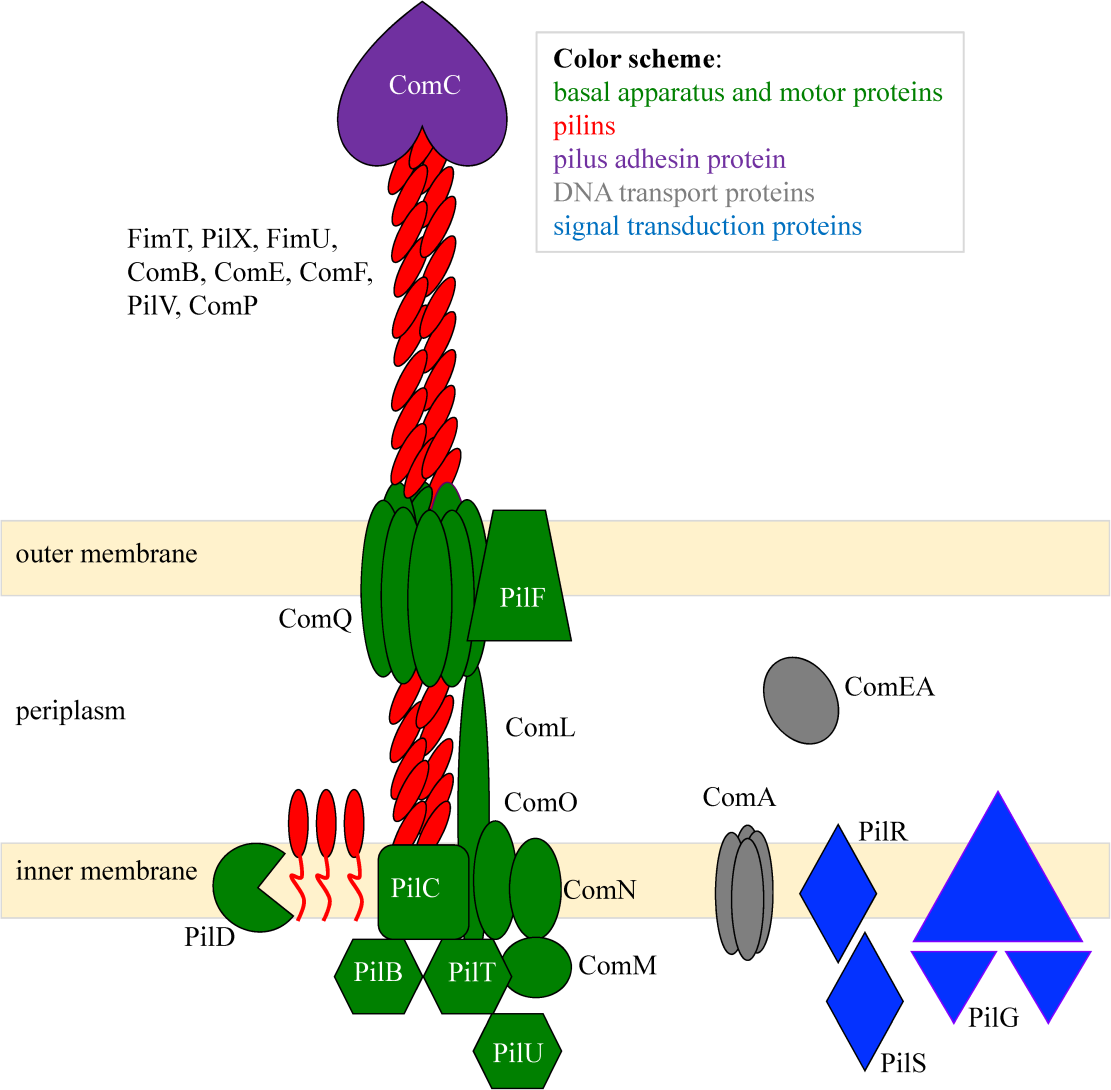
Model of the ADP1 type IV pilus with associated competence and signal transduction proteins. Components of the model are predicted by homology from Table 1 and are supported by data in Fig 3.

The homologous protein functionalities taken together with the known structures of type IV pilus machines in the other bacteria allow us to construct a diagram of the type IV pilus nanomachine in *A*. *baylyi* as shown in Fig 1 [23. 29].

**Fig 2:**
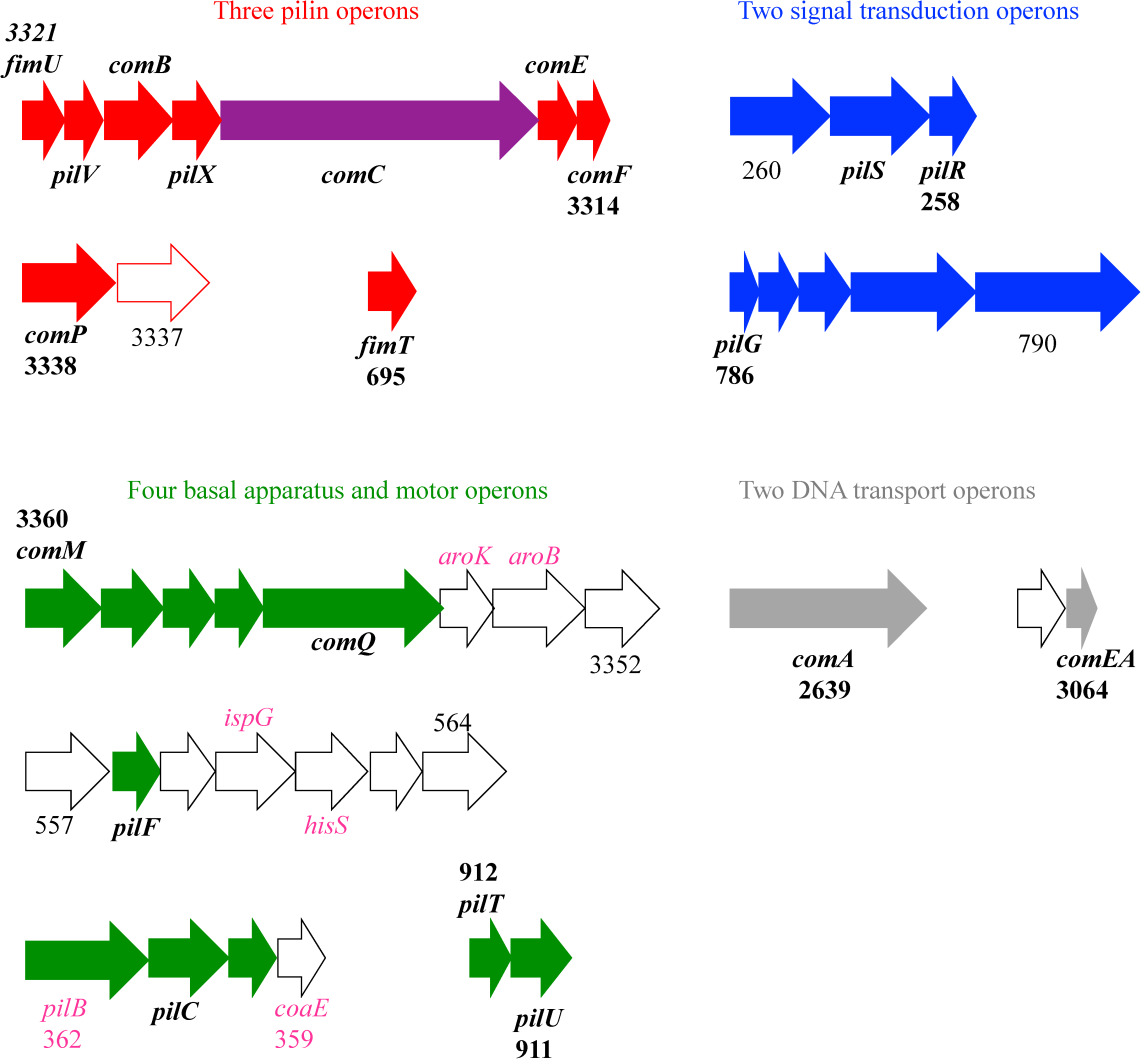
Operons tested in this work. Genes are represented by arrows indicating their approximate length and color-coded according to the general function of the proteins they encode using the same color scheme as in Figs 1 and 3. Red = pilin; purple = adhesin; green = basal apparatus and extension/retraction motors; blue = signal transduction; grey = competence-specific DNA transport; white indicates no known twitching or competence-associated function. To help orient relative to the genome annotation, the ACIAD number for the first and last genes in each operon has been indicated. Gene 3337 has been outlined in red for although it does not encode a pilin, it encodes a glycosylltransferase that modifies ComP [63, 64]. Genes tested in this work are labeled in boldface italics. Essential genes are indicated by pink names.

After identifying the candidate proteins, we identified the operon encoding each. To do this we used software developed by Price [52, 53]. In this tool operons are predicted based on the strand on which two adjacent genes are encoded, the distance between genes in nucleotides, whether the genes are conserved and near each other in multiple genomes, the correlation in gene expression data, whether they share a narrow gene ontology category, and whether they share a Cluster of Orthologous Groups (COG) functional category. To confirm the operons we used the Gene Cluster algorithm in KEGG. In this tool operons are suggested by conserved groups of genes found in a similar order across multiple genomes. The Gene Cluster results agreed with operons identified by the Price methodology. Based on these results, the type IV pilus, competence, and twitching genes are distributed among eleven operons as reported in Tables 1 and 2 and shown schematically in Fig 2.

As seen in Fig 2, the eleven operons encoding potential twitching and competence genes are encoded all over the chromosome. The pilin genes in particular are scattered among three operons. The operons are not preceded by any common repeated sequences or other motifs that suggest co-regulation by a shared activator or repressor, and nor is any subset of them. About half of them are encoded on the leading strand for DNA replication. For each of the type IV pilus operons in Table 1, we referred to a genome-scale knockout screen to list the essential genes in the operons [45]. The essential genes include ones that are needed for isoprenoid synthesis (*ispG*), amino acid synthesis (*aroB* and *aroK*), protein synthesis (*hisS*), central metabolism (coenzyme A synthesis; *coaE*), or pilus extension (*pilB*). None of the proteins in Table 2 are encoded by operons that include essential genes.

### Null mutations in candidate competence and twitching genes

Having identified candidate competence and twitching genes in ADP1 as listed in Tables 1 and 2, we then constructed single-gene deficient mutants to test their phenotypes. The knockouts employed originated from a whole-genome set and are non-polar by design [45]. Several of the knockouts tested (*pilC*, *pilF*, *comQ*, *comM*) were found in operons with downstream essential genes as seen in Fig 2 thus confirming the non-polar nature of the mutations. The mutants were not complemented because many of the type IV proteins were toxic when cloning was attempted, as has been observed in the past [58, 59]. We tested at least one knockout mutation in each of the eleven operons and at least one knockout mutation that would disrupt each of the major sub-assemblies of the type IV pilus such as the assembly platform or the trans-periplasm assemblage that connects the platform to the secretin.

### Competence phenotypes of null mutations in candidate genes

**Fig 3:**
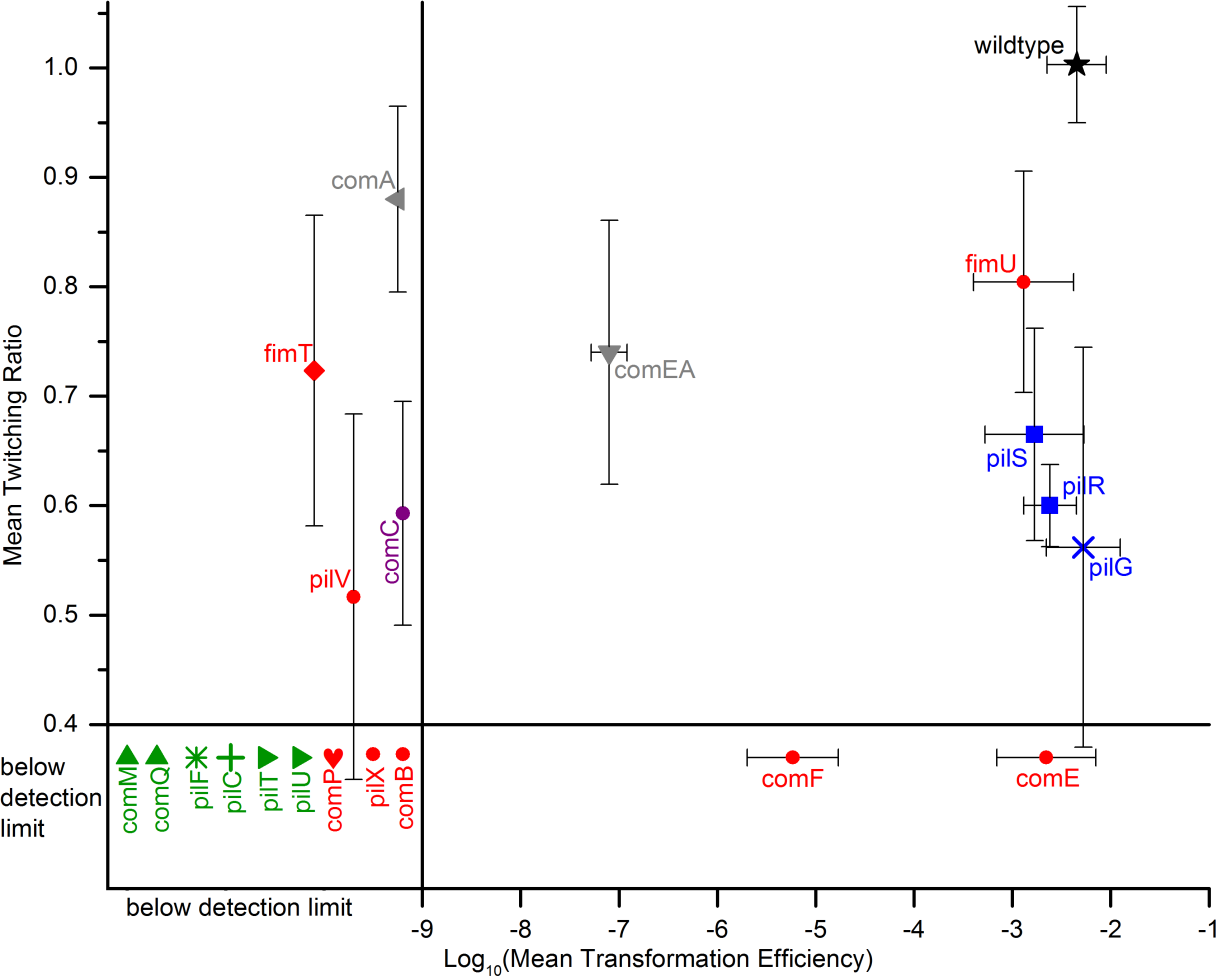
Competence and twitching phenotypes of null mutations. All data in this figure were taken using complex media and incubating at 37°C. Both competence and twitching assays were performed on agar: 1.5% for competence and 0.5% for twitching. Color scheme is the same as Fig 1. Like symbols indicate that genes are part of the same operon. All data points contain multiple measurements for both competence and twitching. Error bars represent the standard deviation of multiple measurements. For the x-axis standard deviation is given by $\Delta x=0.434\frac{\Delta TE}{<TE>}$ where x = log <TE> and ΔTE is defined as the standard deviation from the mean transformation efficiency, <TE>. The detection limit for competence is 10^−9^ and for twitching is 0.4. Data points that fall below either detection limit appear on the graph in a “below detection limit” region. Their position within this region has no physical interpretation beyond indicating that they fall below this limit.

We tested a total of 20 knockout mutants by comparing their transformation efficiency with that of wild type cells. These results are given along the x-axis of Fig 3. We first focus on the 6 data points that report on proteins that comprise the basal structural components of a type IV pilus and its associated motor proteins. These proteins are colored green in Figs 1, 2 and 3. They include an inner membrane assembly platform (PilC), a periplasmic assemblage that connects the inner membrane assembly platform to the secretin (ComM), a secretin (ComQ) and its associated pilotin (PilF) that assists with secretin assembly and localization. Because these four proteins are universal components of a Gram negative type IV pilus, we would expect they are required for competence, and Fig 3 shows they are. Both cytoplasmic pilus retraction ATPases (PilU, PilT) are also required for competence, which could not have been predicted from homology alone because PilT homologs play different roles in type IV pilus phenotypes depending on the organism [65–69].

There are 8 pilins (red) reported on in Fig 3. Since the mechanism of DNA capture in ADP1 is unknown, it is unclear whether the absence of pilins should affect transformation. In agreement with previous results (reviewed in [50]), we found that it does. Although several pilins (ComP, PilV, ComB, PilX, and FimT) were required to observe any transformation, some were not. ComF, for example, is only partially required, while both ComE (a close homolog of ComF [58]) and FimU are not required at all. In the case of FimU, this is in contrast to predictions based on homology. Because of homology to a *P*. *aeruginosa* pilin, we expected that FimU would be required for the assembly of an extracellular pilus, and thus that it would be required for transformation [2]. We discuss this unexpected finding regarding FimU later in this paper. Taken together, the fact that pilins are required for transformation, as shown here and previously, suggests a role for an extracellular pilus in competence in *A*. *baylyi*; however the fact that not all pilins are required for transformation suggests that some pilins may have other specialized functions unrelated to transformation.

The only non-pilin protein encoded by the same operon as *pilV*, *pilX*, *comB*, *comE*, *comF*, and *fimU* is ComC (purple in Fig 3). The *comC* mutant was not competent. This phenotype could not have been predicted from homology alone because ComC homologs are associated with a variety of phenotypes depending on the organism. For example, they have been implicated as adhesins for attachment to biotic surfaces, as necessary for pilus retraction during twitching, or as required for type IV pilus assembly itself, depending on the organism (reviewed in [2]). ComC proteins have been detected in isolated pilus fibers and because of their role in surface adhesion have often been predicted to be at the pilus tip [3, 5, 7, 70–75]. But, a role for ComC proteins in natural transformation appears to be species-specific. *Vibrio cholerae* bacteria apparently do not require ComC for natural transformation because they do not encode a homolog. *N*. *gonorrhoeae*, in contrast, requires a ComC homolog for natural transformation [76].

The *pilR*, *pilS*, and *pilG* genes (blue in Fig 3) were selected for investigation because they regulate type IV pilus function in *P*. *aeruginosa*, where the pilus is used only for twitching since these bacteria are not competent. They may affect the frequency and/or directionality of pilus movement [62]. Although regulation of motor proteins might have been important for competence in *A*. *baylyi*, these genes have no apparent effects on transformation in ADP1.

Finally we consider the two DNA transport proteins, ComA and ComEA (grey). ComA is predicted to encode an inner membrane protein that serves as a channel for single stranded DNA to pass from the periplasm into the cytoplasm. Based on this functionality we expect the *comA* mutant to be not transformable, and that is what we found in agreement with previous results [77]. ComEA homologs are periplasmic proteins needed for maximum transformation in genera such as *Neisseria* and *Vibrio* [28, 31, 37]. While deletion of the *comEA* homolog from *Neisseria* has a mild effect on transformation efficiency, such deletion in *Vibrio* has a dramatic effect [28–31, 37]. Based on this we expect some effect on the transformation efficiency in *A*. *baylyi*, and we find an intermediate one. Based on its homology the mechanism for this effect could be that ComEA binds to DNA in the periplasm thereby trapping it there and increasing the chance that the DNA will encounter the ComA channel and enter the cytoplasm [36]. The residual transformation efficiency of a *comEA* knockout in ADP1 may be due to chance encounters of the DNA with ComA in the absence of the ComEA binding to bias the random walk of the DNA, especially if cytoplasmic recombination-related proteins are abundant and assist in moving the DNA into the cell.

Another observation from Fig 3 is that knockouts in the *fimU pilV comB pilX comCEF* operon (ACIAD3321-3314) do not all have the same natural transformation phenotype, further demonstrating that the insertion cassette is non-polar [45]. For example the upstream *comE* mutant has a higher transformation efficiency than the downstream *comF* mutant, indicating that the *comE* mutation is not polar on *comF*. By similar reasoning, the *fimU* mutation is not polar either, which has already been established by [45].

Some of the *A*. *baylyi* genes we tested (*comP*, *comC*, *comE*, *comF*, *comB*, *pilX* and *comA*) had been previously tested for competence using different mutations and a different methodology: transformation in liquid at 30°C with minimal succinate broth [58, 59, 77–79]. Despite the different methodologies and different null mutations, all but one of our results are the same as those published previously. The exception is *pilX*; the non-polar mutant we tested [45] is not competent while a previous publication reported that a mutation in *pilX* caused a 100-fold loss in transformation efficiency [59].

### Twitching phenotypes of null mutations in candidate genes

**Fig 4:**
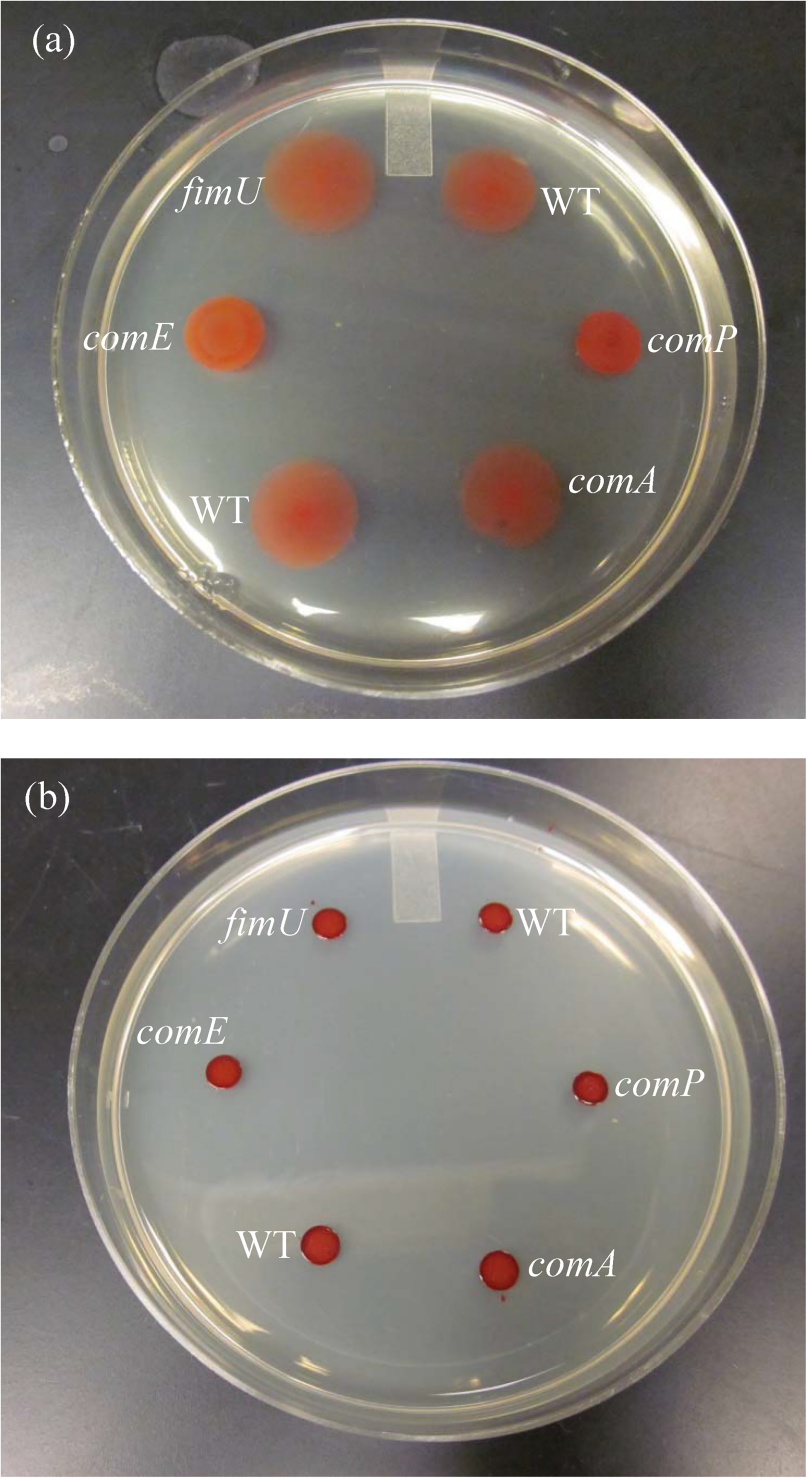
**Twitching zones on (a) soft vs. (b) hard agar for multiple twitching phenotypes.** On soft agar (0.5%) in panel (a), mutants *fimU* and *comA* twitch a comparable amount to the wildtype, while *comP* and *comE* are substantially impaired in agreement with Fig 3. Panel (b) shows the same mutants but now on hard agar (1.5%). In this case all diameters are comparable.

Fig 4(A) shows an example twitch plate with multiple twitching phenotypes displayed. As described in detail in the methods section, we tested twitching by using a soft 0.5% agar surface made with LB and a redox-sensitive dye to improve contrast between the cells and agar. We used exactly the same temperature and nutritional conditions for testing natural transformation and twitching. A twitching ratio is found by measuring the diameter of the mutant’s twitch zone and dividing by the diameter of wild type twitch zone with mutants and wild types growing at the same time on the same batch of plates. It is clear from the figure that the *comA* and *fimU* mutants have similar twitching phenotypes to the wildtype, whereas the *comE* and *comP* mutants have substantial twitching impairments. The *comA* result agrees with previous work [77]. The *comE* and *comP* results disagree with previous work in which these mutants twitched the same as the wildtype when measured on 2% hard agar [58, 79]. Fig 4(B) provides a reason for these differing results.

As shown in Fig 4(B), on 1.5% hard agar the 4 μl inoculum spreads a small distance as the liquid pools before the water absorbs into the plate. All the differentiated twitching phenotypes seen in 4(a) show undifferentiated sized regions in 4(b), thus confirming that the cells cannot twitch on hard agar. We use this fact to find the lowest detectable twitch ratio. We spotted wild type cells on soft and hard agar. On soft agar we let the wildtype cells twitch to a minimum 17 mm diameter because this is the minimum wild type twitch diameter used in any mutant trial. At that time we measured the wild type diameter on hard agar. We divide the hard agar diameter by 17 mm yielding a detection limit of 0.4.

We now focus on the y-axis of Fig 3 to consider the twitching phenotypes of the knockout mutants. Looking at Fig 3, we focus first on the four green data points that report on the basal structural components of a type IV pilus (ComQ, ComM, PilF, and PilC). Given their role in constructing the core type IV pilus machinery, we expected all six of these to be required for twitching, and indeed they are. Both of the pilus retraction ATPases (PilT and PilU) are also required for twitching.

There are 8 pilins (red) reported on in Fig 3. Given that twitching is known to require a pilus, we expected the pilins to be required for twitching. Indeed many of the pilins (ComP, PilX, ComB, ComF and ComE) are required for twitching; however there are several pilins that are not essential to twitching (PilV, and especially FimT and FimU). This suggests an alternative use for pilins by this machine, a topic that will be explored further later.

ComC is encoded by an operon with pilins, so we discuss it here. The *comC* mutant had diminished twitching motility. In some cells, a ComC homolog is required for assembly and/or retraction of the type IV pilus (reviewed in [2]). But in ADP1, ComC is apparently dispensable for assembly and/or retraction of the pilus, or else the cells would not be able to twitch at all. At 1,450 amino acids in length, the ADP1 ComC homolog is much larger than the homologs from *Neisseria* (PilC1 and PilC2; about 1,000 amino acids) or *Pseudomonas* (PilY1; about 1,160 amino acids long), which may account for these functional differences. Although PilC1, PilC2, and PilY1 have regions of similarity along their whole length, the ADP1 homolog ComC is only similar to these in the predicted beta-propeller domain.

Deletion of the signal transduction *pilR*, *pilS*, and *pilG* genes (blue in Fig 3) resulted in reduced twitching. In *P*. *aeruginosa*, the PilS-PilR two-component system regulates transcription of the major pilin. Deletion of *pilR* or *pilS* reduces expression of the major pilin to basal levels [80]. We therefore expected deletion of *pilR* or *pilS* to reduce twitching motility in ADP1, which it does, although only partially, suggesting that PilR/PilS are not the only factors regulating twitching pilus production in ADP1. In *P*. *aeruginosa*, PilG regulates pilus extension so deletion of *pilG* causes a severe loss of piliation and twitching motility [62]. Given this we expected the *pilG* mutant in ADP1 to have reduced twitching motility, which it does. But as with deletion of *pilS* or *pilR*, the phenotype is milder in ADP1 compared with *P*. *aeruginosa*.

Finally we consider the two DNA transport proteins, ComA and ComEA (grey). Since these proteins are associated only with competence in *N*. *gonorrhoeae* and *V*. *cholerae*, we do not expect them to affect twitching. The *comA* mutant behaves as expected, while the *comEA* mutant shows slightly diminished twitching motility. This phenotype was unexpected based on the phenotype of a knockout in *Neisseria* [31]. However, in *Acinetobacter baumannii*, transformation occurs preferentially during twitching [35], indicating there may be some mechanism that connects DNA import and twitching motility in *Acinetobacter*. So we speculate that the *comEA* deletion might affect twitching in *A*. *baylyi* because twitching and competence are closely physiologically intertwined in the genus, at least when the cells are associated with a surface.

Some of the *A*. *baylyi* genes we studied (*comP*, *comC*, *comE*, *comF*, *comB*, *comA*) have been previously tested for twitching motility using different mutations and a different methodology- twitching from streaks made on 2% LB agar at 30°C [58, 59, 77–79]. These papers reported that all mutants twitched the same amount as compared to the wildtype. In contrast on 0.5% soft agar we found varying degrees of twitching impairment from none (*comA*) to intermediate impairment (*comC*) to entirely impaired (*comP*, *comE*, *comF*, and *comB*) as shown in Figs 3 and 4(A). There was no quantification or photographs of previous results to allow for a detailed comparison with our results; however Fig 4 demonstrates that the difference in methodology between using hard (previous results) and soft (present results) agar explains these discrepancies. We did not observe twitching motility by wild type or any mutants on hard agar (≥1.5%) under any environmental or nutritional conditions as shown in Fig 4(B). Using a liquid inoculum, observed spreading on hard agar is due to pooling of the liquid on the agar before it is absorbed, and therefore the same size pool is observed independent of the cells in the pool. In contrast Fig 4(A) shows differentiated twitching zones for those same strains on soft agar.

### Homolog case studies

Many of the proteins listed in Tables 1 and 2 have close, full-length homologs elsewhere in the ADP1 genome. We define close, full-length homologs as ones that have the same predicted conserved domains or protein motifs according to the KEGG database, which relies on multiple sequence alignments and the Pfam, PROSITE, and INTERPRO databases. These homologs are given in Table 3. Two sets of homologs that present interesting case studies are discussed below.

**Table 3:** Full-length homologs of competence and twitching proteins encoded by the ADP1 genome.

Homolog pair	Proteins in the pair	SW-Score^a^	Identity
1	ComF (3314^b^), ComE (3315)	214	0.34
2	FimU (695), FimT (3321)	137	0.30
3	PilB (362), ACIAD3024	1183	0.41
4	PilB (362), ACIAD2241	1124	0.36
5	PilC (361), ACIAD0411	683	0.30
6	PilG (786), PilH (787)	275	0.32
7	PilU (911), PilT (912)	820	0.38

^a^Smith-Waterman algorithm score [81]

^b^Parenthetical numbers refer to the ACIAD genome coordinates.

**Fig 5:**
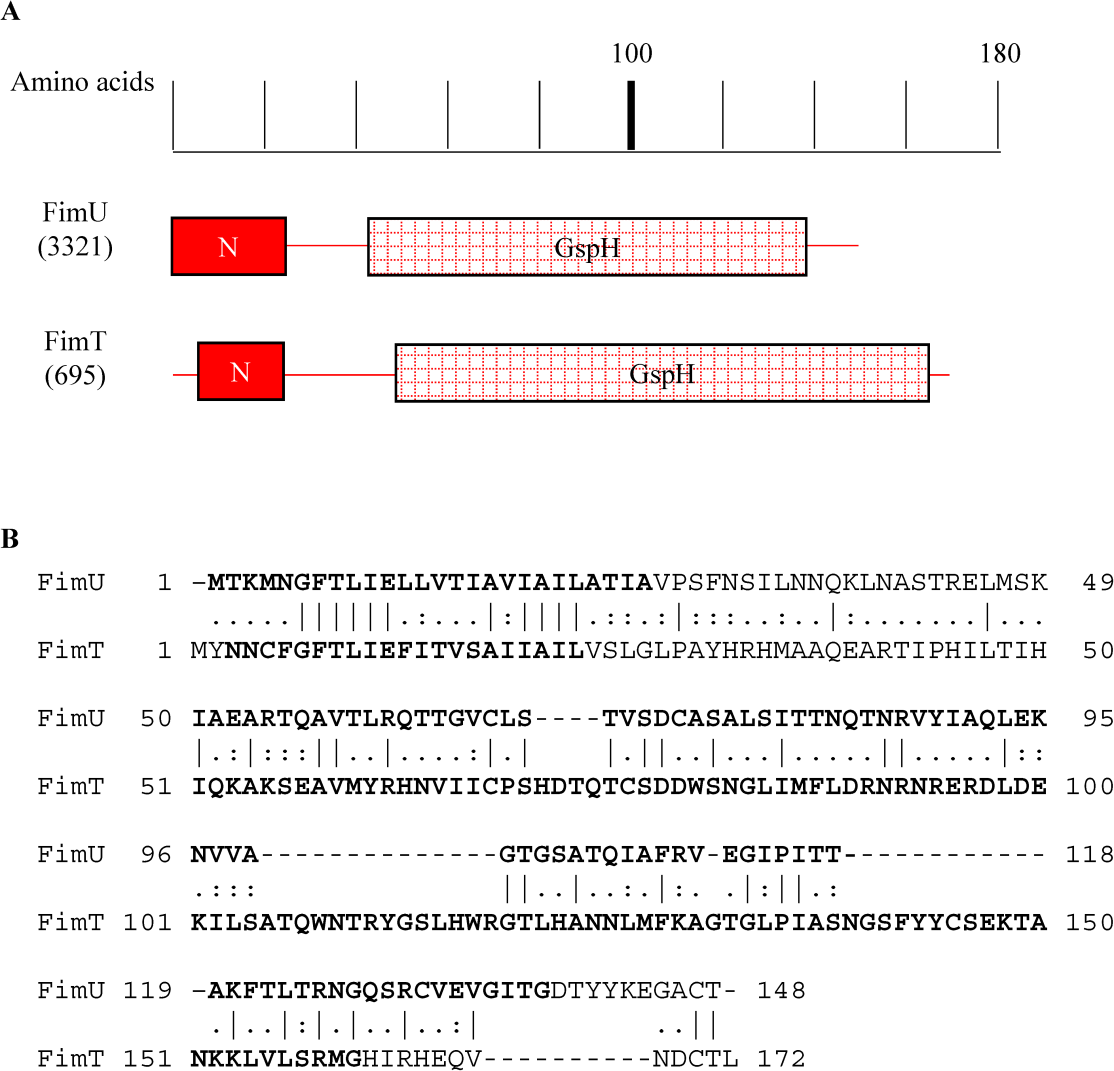
Alignment of FimU and FimT pilins from ADP1. A. The length of the primary sequence is indicated above with Pfam motifs found in both proteins diagrammed below. N = PF07963.11; GspH = PF12019.7. Such motifs are identified using multiple sequence alignments. B. Pairwise alignment of FimU and FimT. Identical amino acids are indicated by a vertical line connecting them while: and. represent higher and lower degrees of chemical similarity between the two amino acids, respectively. Boldface amino acids are part of the Pfam motifs diagrammed in A.

We consider first the case of homologs FimU and FimT. FimU homologs are core minor pilins, required in small amounts for the structure of an extracellular pilus [74], so we expected that FimU would be required for competence and/or twitching as other such pilins are. Contrary to expectation, FimU is not required for competence or twitching. Fig 3 together with Table 3 may provide an explanation. They show that while FimU is not needed for competence, its homolog FimT is. In addition Fig 5 shows a sequence alignment between FimU and FimT. Both proteins have a GspH motif and are especially similar to each other for the first 100 amino acids. Taken together, their overlapping structures and their phenotypes as given in Fig 3, suggests that some aspect of FimU’s functionality is for transformation, and in FimU’s absence its homolog FimT can substitute for it. The evolutionary histories of FimU and FimT, described next, support this suggestion.

Their operon structure suggests that the two genes encoding FimU and FimT have different evolutionary histories. FimT is encoded by a monocistronic operon unlinked to the polycistronic operon encoding FimU and other pilins PilV, ComB, PilX, ComE, and ComF. Because the genes are unlinked they likely have different origins in the ADP1 genome. Indeed using the Gene Cluster tool in KEGG, we discovered that *fimT* is part of a cluster found only in the same family (*Moraxellaceae*) as *A*. *baylyi*, whereas *fimU* is part of a cluster of pilin genes that have conserved gene order in many more organisms, including distant relatives. FimT thus appears to have a more recent origin in the *Moraxellaceae* than FimU. So we posit that the function of a FimU/T homolog is in fact required for type IV pili and in ADP1 the latecomer homolog FimT can substitute for FimU as needed.

**Fig 6:**
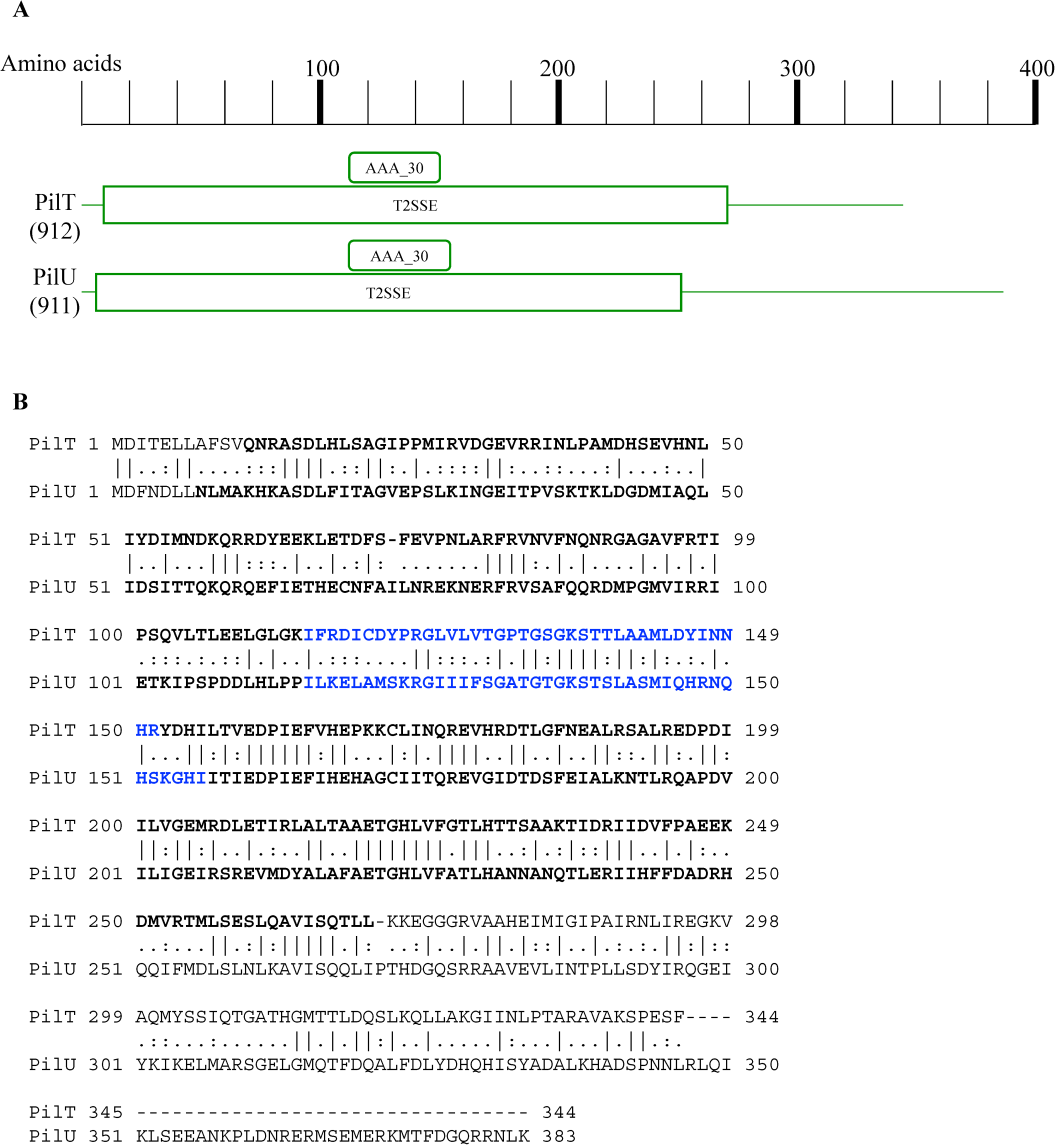
Alignment of PilT and PilU retraction motors from ADP1. A. The length of the primary sequence is indicated above with Pfam motifs found in both proteins diagrammed below. T2SSE = Type II/IV secretion system protein; AAA_30 = AAA_proteins. AAA_30 domains contain a P-loop motif with Walker A and Walker B motifs. Such motifs are identified using multiple sequence alignments. B. Pairwise alignment of PilT and PilU. Identical amino acids are indicated by a vertical line connecting them while: and. represent higher and lower degrees of chemical similarity between the two amino acids, respectively. Boldface amino acids are part of the Pfam motifs diagrammed in A while the blue text indicates more specifically the AAA_30 motifs.

We now consider the retraction motor proteins PilU and PilT which are close homologs of each other (Fig 6). Many organisms have multiple PilT homologs and their function, where known, is species-specific [65–69]. For example in *N*. *gonorrhoeae*, there are three closely related homologs PilT, PilT2, and PilU. Deletion of each one has different effects. Deletion of *pilT* prevents pilus retraction [66]; deletion of *pilU* has little effect on twitching motility, and deletion of *pilT2* causes a 2-fold decrease in the speed of twitching motility [65]. In ADP1, there are two possible type IV pilus retraction motors: PilT and PilU. They are encoded in the same operon and both are required for competence and for twitching. This fact implies they cannot substitute for each other and therefore have unique functionalities like the PilT homologs in *N*. *gonorrhoeae* discussed above. To explore a structural explanation, we used protein alignment to examine their similarities and differences (Fig 6). We find that PilT and PilU are similar to one another along their whole lengths except that PilU is longer with a 37-amino acid extension at the C-terminal end. This C-terminal region is rich in charged amino acids such as R and K (10, or 27% of the amino acids) and E (5, or 13% of the amino acids). The unique C-terminal ends may account for why PilT and PilU are not interchangeable and are both necessary for both twitching and transformation.

### Interrelationship of competence and twitching

Looking at Fig 3, we now consider the interrelationship between the transformation and twitching phenotypes. A striking observation is that 9 of the 20 genes tested are found in the lower left corner of the graph, including all that form the basal type IV pilus machine (green data points) along with the pilins, ComP, ComB and PilX (red). This position on the graph indicates that these genes are required for both transformation and twitching. Therefore these proteins are likely required for type IV pilus assembly irrespective of the function of that pilus. These pilins should therefore be considered the core pilins in ADP1 and likely play an essential role in pilus assembly or in an activity common to both twitching and natural transformation, such as extension or retraction (reviewed in [2]). Further these observations imply that the molecular basis of both twitching and natural transformation in ADP1 involves a shared type IV pilus comprised of the same core components.

Another striking observation from Fig 3 is that mutants that are not in the lower left corner are located mostly on the periphery of the graph. The middle of the graph is mostly empty. This implies that most proteins not required for both transformation and twitching, have specialized in either transformation or twitching. For example and as expected, ComA, the protein that transports DNA across the inner membrane, specializes in transformation, while the PilG, PilS, and PilR signal transduction proteins specialize in twitching. We refer to proteins specialized in one phenotype as accessory proteins in contrast to the core type IV pilus proteins needed for both twitching and natural transformation.

The pilins (red) are particularly varied in their specialization. Mutants lacking a single pilin are located in every region of Fig 3. As discussed earlier, three of the ADP1 pilins (ComP, ComB, and PilX) are required for both twitching and transformation, suggesting that they are universal components of the type IV pilus irrespective of its function. Other pilins are specialized for either transformation (FimT) or twitching (ComE). ComF is an accessory that enhances transformation but is absolutely required for twitching. The FimU pilin is not needed for either transformation or twitching and could therefore be used for another functionality entirely or, given the arguments above regarding its homolog FimT, may be involved in transformation.

**Fig 7:**
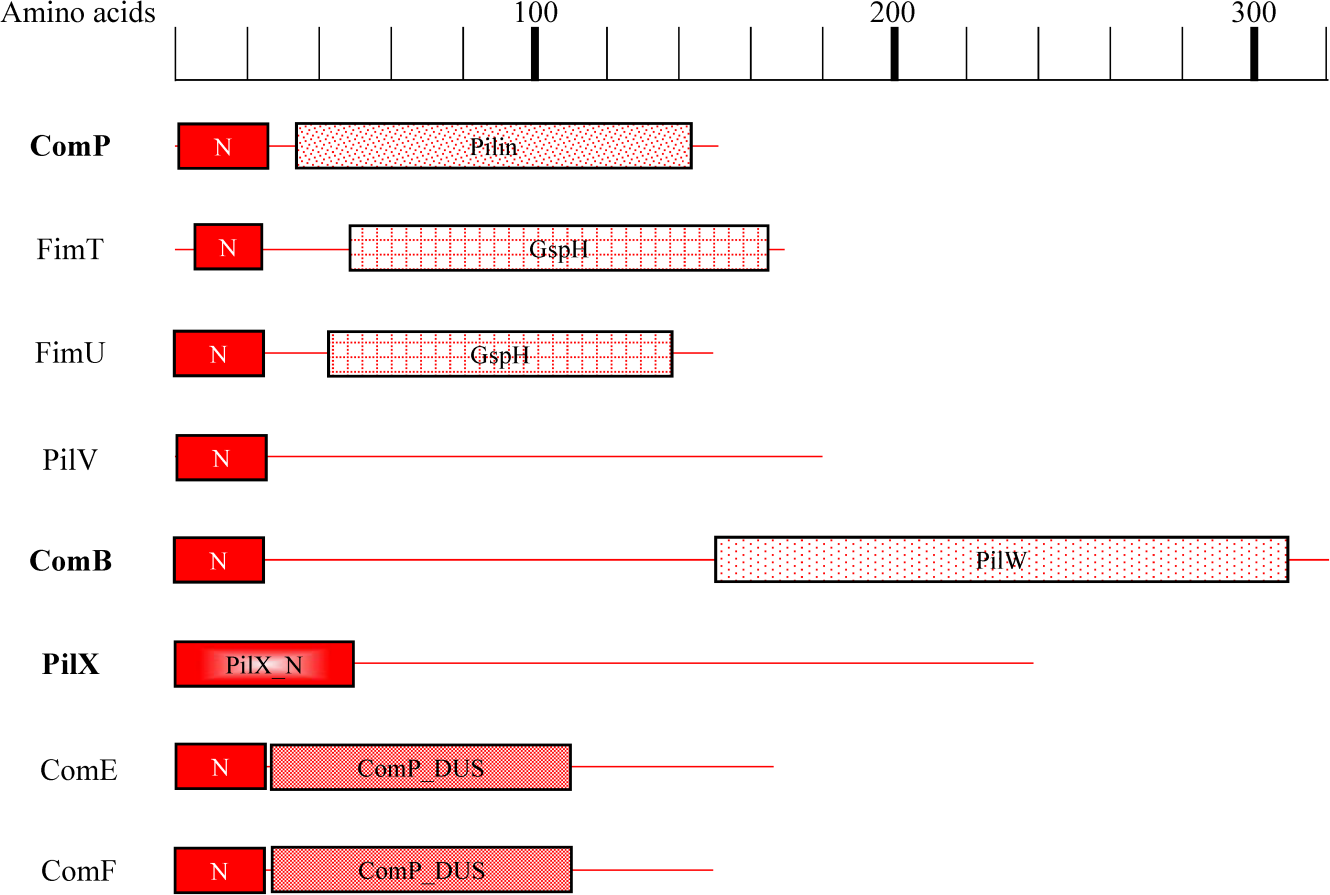
Protein motif diagrams of the type IV pilin proteins encoded by ADP1. Length in amino acids is indicated along the top, with pilins diagrammed below. Pilins absolutely required for both twitching and competence are in boldface. Pfam motifs are indicated by filled, labeled boxes. The Pfam motif names are: N = PF07963.11 Prokaryotic N-terminal methylation motif; Pilin = PF00114.18 Pilin (bacterial filament); GspH = PF12019.7 Type II transport protein GspH; PilW = PF16074.4 Type IV Pilus-assembly protein W; PilX_N = PF14341.5 PilX N-terminal; ComP_DUS = PF16732.4 Type IV minor pilin ComP, DNA uptake sequence receptor.

The varied functionalities for the pilins is supported by Fig 7 which shows the similarities and differences in the pilins’ identifiable protein motifs. All the pilins are similar in their N-termini regions. This similarity indicates they are all targets for the same peptidase (PilD), and that they all can be incorporated into the pilus filament. However, despite the similarities in their N termini regions, the pilins are very dissimilar along the rest of their lengths and in fact cannot be aligned as a group. These differences are the structural basis for their different specializations in agreement with the varied locations of the pilin mutant phenotypes in Fig 3.

## Discussion

In this paper we used bioinformatics to identify potential natural transformation and twitching genes and their operons in the bacterium *Acinetobacter baylyi*. We then measured the transformation efficiency and twitching phenotypes of the bioinformatically-identified genes. Although competence and twitching are two bacterial functionalities that on their surface appear dissimilar, the results presented here show that natural transformation and twitching in *A*. *baylyi* both utilize the same core type IV pilus proteins. The core includes the inner membrane assembly platform (PilC), a periplasmic assemblage connecting the inner membrane platform to the secretin (ComM), a secretin (ComQ) and its associated pilotin (PilF) that assists with secretin assembly and localization, both cytoplasmic pilus retraction ATPases (PilU, PilT), and three pilins (ComP, ComB, and PilX). We also find that these required proteins are encoded in 6 different unlinked operons.

In this paper we also found that proteins not needed for both competence and twitching are instead found to specialize in one or the other. The pilins studied herein (FimT, PilV, ComP, PilX, ComB, ComF, ComE, and FimU) are particularly varied in their specialization with some specialized for transformation (FimT), others for twitching (ComE), and some required for both (ComP, PilX, ComB). In addition we find these pilins are dissimilar along most of their length. Nonetheless all pilins are found to be similar in their N termini regions and therefore can be used in filaments attached to the same basal machine. These findings allow us to address the nature of the transformation and twitching nanomachines.

Some have argued that in some species twitching, but not natural transformation, nanomachines involve extracellular pili despite the involvement of type IV pilus genes in both phenotypes [23, 82]. However, short extracellular transformation pili were recently detected in *Vibrio cholerae* and *Streptococcus* [27, 29]. In *Acinetobacter baumannii*, twitching and competence are physiologically linked so that many isolates are naturally transformable only while they are twitching [35]. These findings and the results presented here suggest a model for twitching and competence in ADP1. Given the overlap of required genes for competence and twitching shown in Fig 3, it appears that both competence and twitching require use of the same type IV pilus basal apparatus. Since both functionalities require multiple pilins as well as both pilus retraction motors PilU and PilT, we suggest that both functionalities make use of a pilus protruding from this nanomachine. However given that competence and twitching require a different, yet overlapping, set of pilins and that those pilins have unique amino acid sequences, this suggests that the competence and twitching pili employ a distinct set of pilins to create different pili specialized for each functionality, or that incorporation of different pilins in the same fiber allows a single appendage to carry out both transformation and twitching.

Species lacking certain key pilin or other accessory proteins required for natural transformation may not then be competent, and likewise for twitching. Thus, differences in competence and twitching phenotypes between species that encode core type IV pilus genes may arise from variations in which pilins or other accessory proteins are encoded in their genomes and under what conditions those proteins are expressed.

## Supporting information

S1 Table (DOCX)
