# Supplementary material for: The role of core and accessory type IV pilus genes in natural transformation and twitching motility in the bacterium Acinetobacter baylyi
Source: PLoS One. 2017 Aug 3;12(8):e0182139. doi: 10.1371/journal.pone.0182139 (PMC5542475; doi:10.1371/journal.pone.0182139)
Supplement: S1 Table — (DOCX) [file pone.0182139.s001.docx]

**Supporting Information**

**S1 Table: Strain table**

| Name | Strain catalog number |
| --- | --- |
| ADP1^a^ | CCL2055 |
| *pilC::tdk-kan^b^* | CCL2069 |
| *pilF::tdk-kan* | CCL2077 |
| *fimT::tdk-kan* | CCL2079 |
| *pilU::tdk-kan* | CCL2131 |
| *pilT::tdk-kan* | CCL2133 |
| *comF::tdk-kan* | CCL2111 |
| *comE::tdk-kan* | CCL2113 |
| *comC::tdk-kan* | CCL2135 |
| *pilX::tdk-kan* | CCL1914 |
| *comB::tdk-kan* | CCL1784 |
| *pilV::tdk-kan* | CCL2115 |
| *fimU::tdk-kan* | CCL2147 |
| *comP::tdk-kan* | CCL1780 |
| *comQ::tdk-kan* | CCL1788 |
| *comM::tdk-kan* | CCL2119 |
| *comA::tdk-kan* | CCL2107 |
| *comEA::tdk-kan* | CCL2109 |
| *pilR::tdk-kan* | CCL2065 |
| *pilS::tdk-kan* | CCL2067 |
| *pilG::tdk-kan* | CCL2081 |
|  |  |
| ^a^From American Type Culture Collection ATCC33305 | |
| ^b^Mutations obtained from [45] | |
